# Draft Genome Sequence of *Chelatococcus sambhunathii* Strain HT4^T^ (DSM 18167^T^) Isolated from a Hot Spring in India

**DOI:** 10.1128/genomeA.00825-16

**Published:** 2016-08-11

**Authors:** Jhasketan Badhai, William B. Whitman, Subrata K. Das

**Affiliations:** a Department of Biotechnology, Institute of Life Sciences, Nalco Square, Bhubaneswar, India; b Department of Microbiology, University of Georgia, Athens, Georgia, USA

## Abstract

The moderately thermophilic bacterium *Chelatococcus sambhunathii* strain HT4^T^ was isolated from hot spring sediment. Based upon the draft genome sequence, the genome is 4.4 Mb and encodes 4,147 proteins.

## GENOME ANNOUNCEMENT

*Chelatococcus sambhunathii* strain HT4^T^ is a Gram-negative, aerobic rod. It is nonsporulating and motile, belonging to the class *Alphaproteobacteria*. It was isolated from a hot spring located in Athamallik, Orissa, India (1). It grows heterotrophically at pH 6.0 to 8.5 and at 20 to 50°C.

The draft genome of *C. sambhunathii* strain HT4^T^ was generated at the DOE Joint Genome Institute (JGI) using the Illumina HiSeq 2000 platform (2). An Illumina standard shotgun library was constructed and sequenced using the Illumina HiSeq 2000 platform, which generated 8,684,234 reads totaling 1,311.3 Mb. The filtered Illumina reads were assembled using Velvet (3), wgsim (https://github.com/lh3/wgsim), and Allpaths-LG (4) tools. The final draft assembly contains 38 contigs in 35 scaffolds, totaling 4.4 Mb, with an input read coverage of 193.7-fold. The largest and *N*_50_ contigs are 742.1 kb and 426.1 kb, respectively, with a G+C content of 67.8%.

The genome was annotated using the JGI Microbial Genome Annotation Pipeline (5). Genes were identified using the Prodigal (6) and GenePRIMP (7) programs. The tRNAs, rRNAs, and other noncoding RNA genes were identified by searching the genome using the tRNAScan-SE tool (8), rRNA gene models built from SILVA (9), and Infernal (http://infernal.janelia.org), respectively. The draft genome contains 4,147 coding sequences, 49 tRNAs, 4 rRNAs, 13 other RNAs, and two clustered regularly interspaced short palindromic repeats.

### Accession number(s).

This whole-genome shotgun project has been deposited at DDBJ/EMBL/GenBank under the accession number LIOL00000000. The version described in this paper is version LIOL01000000.
